# A latent profile analysis of self-regulatory fatigue and its relationship with activation in patients receiving chemotherapy for breast carcinoma: An observational study

**DOI:** 10.1097/MD.0000000000042398

**Published:** 2025-05-16

**Authors:** Ying Yang, Jing Zhao, Yina Liu, Li Li

**Affiliations:** a Nursing Department, North Sichuan Medical College, Nanchong, China.

**Keywords:** breast carcinoma, chemotherapy, latent profile analysis, patient activation, self-regulatory fatigue

## Abstract

This study explores the profiles of self-regulatory fatigue (SRF) in patients with breast cancer who received chemotherapy and their influencing factors and to analyze the relationships between these profiles and patient activation. A total of 315 individuals with breast cancer who received chemotherapy were chosen using convenience sampling between January and April 2024, and a cross-sectional survey was conducted, comprising a self-administered basic information questionnaire, the SRF Scale, and the Patient Activation Measure. SRF profiles were identified utilizing latent profile analysis, and factors that might influence the SRF profiles identified were analyzed employing the chi-square test and multiple regression analysis. Further, differences in activation among patients with the identified SRF profiles were assessed using an analysis of variance. The SRF of patients with mammary carcinoma receiving chemotherapy could be divided into 3 potential profiles: a high SRF, cognitively weakened group (29.2%), a moderate SRF, borderline group (46.7%), and a low SRF, behavioral stabilization group (24.1%). Medical payment method, disease duration, disease stage, number of chemotherapy-related symptoms, and whether or not the patient had undergone surgery for breast cancer were factors associated with patient SRF (*P* < .05). The 3 potential SRF profiles showed notable variations in patient activation levels (*F *= 83.707, *P* < .001). SRF was categorized into 3 profiles in individuals with mammary carcinoma undergoing chemotherapy. Healthcare professionals should focus on patients with low income, long disease duration, advanced disease stage, many chemotherapy-related symptoms, and who have undergone breast cancer surgery. In addition, SRF is closely related to patient activation, suggesting that interventions should be targeted based on these different SRF profiles to improve patient activation.

## 1. Introduction

According to the Global Cancer Statistics report, the tally of newly diagnosed occurrences of breast carcinoma in women reached approximately 2.3 million in 2022, accounting for 11.6% of all new neoplasm cases, second only to lung carcinoma in terms of prevalence.^[1]^ It was also estimated that female breast cancer was among the top 5 malignant tumors in China.^[2]^ Chemotherapy is an important part of breast cancer treatment, as it improves patient survival rates. However, chemotherapy also has side reactions, including gastrointestinal toxicity, cardiotoxicity, peripheral neuropathy, and skin changes.^[3–6]^ Treatment cycles for breast cancer are long, and the toxic side effects of chemotherapy place patients in a state of chronic physical and mental stress. Most patients with breast cancer are women. Influenced by traditional Chinese thinking, women take more responsibility for taking care of their families and family members, and they need to continue to take care of the needs of their families and family members while undergoing treatment, which brings them additional psychological and emotional burdens, depletes their self-regulatory resources, and impairs their self-regulatory ability, which may lead to self-regulatory fatigue (SRF) in such patients. SRF refers to the overconsumption of self-control resources, resulting in decreased self-regulatory ability and a weakened willingness to control oneself.^[7]^ According to the self-control power model,^[8]^ the higher the level of SRF experienced by an individual, the more self-control energy is lost, and the less self-control the person has, where self-control involves the regulation of one’s impulses, desires, and emotions, and is crucial in achieving predetermined goals or conforming to socially normative behaviors.^[9]^

A variety of personal and environmental factors can impose physical and psychological burdens on patients, resulting in a high depletion of self-control resources, difficulty in effectively managing cognition and emotions, and growth of negative thinking, which in turn affect patient activation.^[10]^ Patient activation refers to the extent to which patients are likely to be proactive in self-health management and the cognition, skills, actions, and beliefs they possess for self-health management.^[11]^ Patient activation is crucial for evaluating self-management potential and health outcomes.^[12,13]^ Research has shown that individuals with SRF have reduced amygdala function.^[14]^ The amygdala is responsible for generating, recognizing, and regulating emotions. This indicates that patients with SRF may struggle to control their emotions, which could also impact patients’ motivation for self-management. In October 2023, the Office of Health Emergencies in China emphasized in its document on cancer prevention and control the importance of advocating a healthy lifestyle, spreading health knowledge, and encouraging people to actively participate in cancer prevention and treatment. This highlights the significance of individuals taking the initiative to maintain their own health.^[15]^ Improving patient activation promotes good health behaviors, optimizes disease prognosis, enhances the quality and efficiency of nursing services, and reduces unnecessary medical expenditure.^[16–18]^

To date, studies on SRF have focused on nursing students,^[19]^ patients with coronary heart disease,^[20]^ and individuals undergoing peritoneal dialysis,^[21]^ while there have been few studies on the SRF status of individuals diagnosed with mammary carcinoma and treated with chemotherapy and the factors affecting it. Further, neither the relationship between SRF and patient activation nor the activation characteristics of individuals with different SRF profiles among patients with breast cancer undergoing chemotherapy have been comprehensively explored, and understanding these relationships will be crucial for improving patient SRF, increasing their activation and promoting high quality cancer control and treatment. Existing studies mostly use scale scores as the judgment criterion, with less consideration of inter-individual differences. Latent profile analysis can better understand group heterogeneity by analyzing the scores of scale entries or dimensions, revealing potential characteristics or attributes of the group, and categorizing them. Therefore, this study used latent profile analysis to analyze different categories of SRF in breast cancer patients undergoing chemotherapy, to explore the factors associated with these categories, and to investigate the relationship between SRF and patient activation.

## 2. Materials and methods

### 2.1. Ethical statement and patient consent

The Ethics Committee of the Affiliated Hospital of North Sichuan Medical College has granted its approval for this research (Approval Number: 2024ER119-1). Consent was obtained from all respondents before the study commenced.

### 2.2. Participants

In this study, patients with breast cancer who received chemotherapy from January to April 2024 in 2 tertiary hospitals in Sichuan Province, China, were selected by convenience sampling. Criteria for inclusion were as stated below: individuals diagnosed with mammary carcinoma by pathologic examination, undergoing chemotherapy, normal communication ability, and voluntary engagement in this investigation. Criteria for exclusion were as stated below: additional malignancies coexisting at the same time and mental disorders. The sample size was calculated based on 5 to 10 times the number of variables, with a 20% allowance for ineligible surveys included in the calculation. This investigation included 15 variables (13 general information items, 1 SRF item, and 1 patient activation item), and the necessary sampling range was estimated to vary from 94 to 188 participants. A sum of 320 patients took part in the research and 5 patients withdrew, resulting in the inclusion of 315 eligible patients.

### 2.3. Measures

#### 2.3.1. Basic information questionnaire

This questionnaire was employed to gather sociodemographic and disease-related information from patients. Sociodemographic data included occupational status, marital status, medical payment method, etc. Disease-related information included disease duration, disease stage, number of chemotherapy-related symptoms, presence of chronic disease, and whether or not the patient had undergone surgery for breast cancer.

#### 2.3.2. Self-regulatory fatigue scale

The original scale was developed by Nes.^[7]^ The Chinese version of the scale, translated by Wang,^[22]^ was used in this study. SRF-S contains 3 dimensions, with a total of 16 items, as follows: a cognition dimension (6 items), an emotion dimension (5 items), and a behavior dimension (5 items). The SRF-S assessment uses a 5-point Likert scale, ranging from 1 (strongly disagree) to 5 (strongly agree). A higher score reflects a stronger degree of SRF. In this research, the internal consistency of this scale was measured at 0.906 using Cronbach alpha coefficient.

#### 2.3.3. Patient activation measure

The original PAM scale was developed by Hibbard.^[11]^ The Chinese version of the scale, translated by Hong,^[23]^ was utilized in the investigation. PAM is scored using a Likert 5-point scale, ranging from 0 (not applicable) to 4 (strongly agree), and the raw scores are converted to a scale from 0 to 100. Higher scores reflect a higher level of patient activation. Based on PAM scores, patient activation was categorized into 4 levels, as follows: first level (low level), total score ≤ 47; second level (low and medium level), 47.1 to 55.1; third level (medium level), 55.2 to 67.0; and fourth level (high level), ≥ 67.1. In this research, the internal consistency of this scale was measured at 0.778 using Cronbach alpha coefficient.

### 2.4. Data collection

Data were collected before patients had completed their previous course of chemotherapy and were about to start their next course of chemotherapy drugs. At the time of the survey, patients had completed at least 1 course of chemotherapy. Using an anonymous survey approach, the researcher clarified the aim and specifics of the research to eligible participants and distributed the questionnaire after securing their approval. The researcher explained the questionnaire notes to the respondents with unified instruction, and the respondents completed the questionnaire independently. If the respondents asked for clarification of any entries, they were promptly explained. The researcher collected and checked the questionnaires on the spot and then evaluated them after confirming no errors.

### 2.5. Data analysis

Potential SRF profiles were determined using Mplus 8.3 software, with the 3-dimensional SRF-S scores as indicators. The optimal classification was determined based on a series of fitted information indices, including log-likelihood value, Akaike information criterion (AIC), Bayesian information criterion (BIC), adjusted BIC (aBIC), entropy of information, Bootstrapped Likelihood Ratio Test (BLRT), and Lo-Mendell-Rubin test (LMRT). AIC, BIC, and aBIC values were minimized, and entropy values closest to 1 were selected (where values ≥ 0.8 indicated 90% categorization accuracy), along with LMRT and BLRT reaching the significance level.^[24]^ IBM SPSS 26.0 was used for descriptive statistical analysis. Categorical variables are expressed as frequency (percentage), and continuous variables as mean and standard deviation. The chi-square test was used for the univariate analysis of potential SRF profiles; factors with *P < *.05 in the univariate analysis were included in the multivariate logistic regression analysis. Analysis of variance was used for between-group comparisons of patient activation scores. In this study, differences were considered statistically significant at *P < *.05.

## 3. Results

### 3.1. Common method bias test

In the present research, Harman single-factor test^[25]^ results indicated 7 distinct factors with eigenvalues exceeding 1. Furthermore, the cumulative variance accounted for by the first factor amounted to 29.984%, which fell short of the critical threshold of 40%. These findings suggest that common method bias did not significantly influence the current study.

### 3.2. Potential SRF profiles

**Figure 1.**
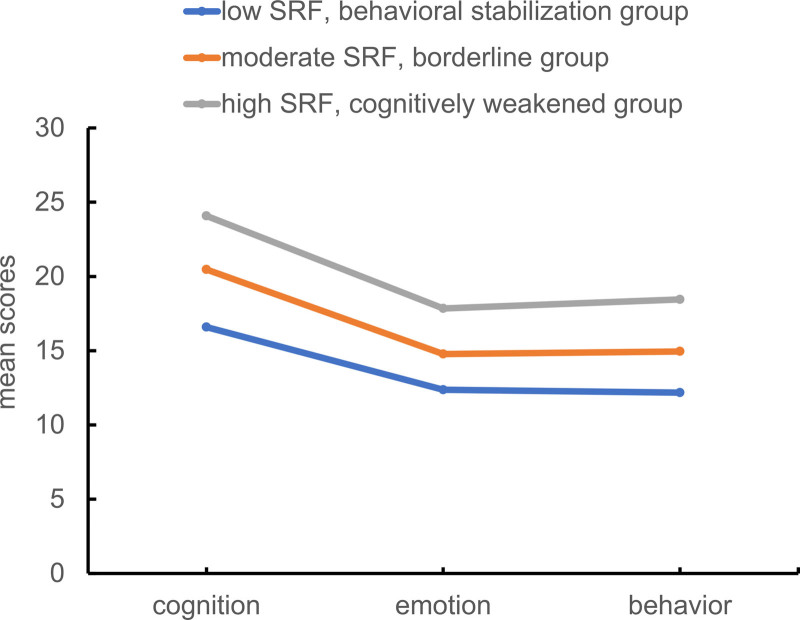
Mean scores of dimensions for SRF profiles. SRF = self-regulatory fatigue.

Latent profile analysis was performed based on the 3 dimensions of SRF-S, 1 to 5 potential profile models were constructed, and the specific results have been detailed in Table 1. In models 1 to 4, the values of AIC, BIC, and aBIC sequentially decreased. However, in model 5, while the values of AIC were decreasing, those of BIC and aBIC appeared to increase, and the *P*-values of LMR and BLRT were not significant (*P* > .05). The AIC, BIC, and aBIC values for Model 4 are smaller than those of AIC, BIC, and aBIC for Model 3, indicating a better model fit for Model 4. However, the entropy value for Model 4 is smaller than that of Model 3, indicating that Model 3 has a higher classification accuracy. According to Table 2, the average probability of belonging to each category in Model 3 is 0.905, 0.952, and 0.941, respectively, indicating that the results are highly reliable. Meanwhile, considering the simplicity of classification, we chose Model 3 as the optimal model. Profile 1 had high scores on all dimensions, with the highest score on the cognitive dimension, therefore it was named the high SRF, cognitively weakened group, a total of 92 cases (29.2%) were included in this profile. Profile 2 included scores between those of profiles 1 and 3, therefore it was named the moderate SRF, borderline group, and included 147 cases (46.7%). Patients with profile 3 had low scores on all dimensions, with the lowest scores on the behavioral dimension, therefore, patients with this profile were referred to as the low SRF, behavioral stabilization group, and comprised 76 cases (24.1%). These results are summarized in Table 3 and Figure 1.

**Table 1:** Results of potential profiles of SRF.

Model	K	Log (L)	AIC	BIC	aBIC	Entropy	LMR	BLRT	Potential profile proportion
1	6	−2297.688	4607.376	4629.892	4610.861	–	–	–	–
2	10	−2093.133	4206.265	4243.791	4212.074	0.839	<0.001	<0.001	0.394/0.606
3	14	−2010.993	4049.985	4102.521	4058.117	0.839	<0.001	<0.001	0.292/0.467/0.241
4	18	−1984.335	4004.670	4072.216	4015.125	0.833	<0.001	<0.001	0.406/0.232/0.257/0.105
5	22	−1979.718	4003.436	4085.993	4016.215	0.775	0.620	0.667	0.225/0.086/0.337/0.251/0.102

aBIC = adjusted Bayesian information criterion, AIC = Akaike information criterion, BIC = Bayesian information criterion, BLRT = Bootstrapped Likelihood Ratio Test, LMR = Lo-Mendell-Rubin test, Log(L) = log-likelihood, SRF = self-regulatory fatigue.

**Table 2:** Average probability of each potential category.

Latent class	1	2	3
1	0.905	0.063	0.032
2	0.048	0.952	<0.001
3	0.059	<0.001	0.941

**Table 3:** Means and standard deviations of the total SRF scores and scores on each dimension for different potential profiles.

	High SRF, cognitively weakened group (n = 92)	Moderate SRF, borderline group (n = 147)	Low SRF, behavioral stabilization group (n = 76)
Total score, M (SD)	60.37 (3.46)	50.19 (2.79)	41.13 (2.68)
Cognitive, M (SD)	24.08 (1.92)	20.47 (1.69)	16.59 (1.47)
Emotional, M (SD)	17.85 (1.27)	14.77 (1.22)	12.37 (1.19)
Behavioral, M (SD)	18.45 (1.68)	14.95 (1.57)	12.17 (1.16)

SRF = self-regulatory fatigue.

### 3.3. Univariate analysis of factors associated with SRF profiles

Univariate analysis showed statistically significant differences among patients with the 3 SRF profiles in terms of age, monthly income (Chinese Yuan), medical payment method, disease duration, disease stage, number of chemotherapy-related symptoms, and whether or not they had undergone surgery for breast cancer (*P* < .05) (Table 4).

**Table 4:** Univariate analysis of factors associated with SRF profiles.

Variables	High SRF, cognitively weakened group (n = 92)	Moderate SRF, borderline group (n = 147)	Low SRF, behavioral stabilization group (n = 76)	χ^2^	*P*
Age (yr)					
18 to 45	12 (13.0%)	27 (18.4%)	24 (31.6%)	9.396	.009
≥45	80 (87.0%)	120 (81.6%)	52 (68.4%)		
Place of residence					
Rural	53 (57.6%)	68 (46.3%)	45 (59.2%)	4.629	.099
Urban	39 (42.4%)	79 (53.7%)	31 (40.8%)		
Occupational status					
Employed	60 (65.2%)	91 (61.9%)	51 (67.1%)	0.656	.720
Unemployed	32 (34.8%)	56 (38.1%)	25 (32.9%)		
Marital status					
Others	5 (5.4%)	17 (11.6%)	8 (10.5)	2.584	.275
Married	87 (94.6%)	130 (88.4%)	68 (89.5%)		
Education level					
Primary school	35 (38.0%)	59 (40.1%)	25 (32.9%)	2.846	.828
Junior high school	37 (40.2%)	49 (33.3%)	28 (36.8%)		
Senior high school	15 (16.3%)	27 (18.4%)	16 (21.1%)		
College and above	5 (5.4%)	12 (8.2%)	7 (9.2%)		
Monthly income (Chinese Yuan)					
≤2000	35 (38.0%)	21 (14.3%)	8 (10.5%)	49.596	<.001
2001 to 3000	34 (37.0%)	57 (38.8%)	14 (18.4%)		
3001 to 4000	18 (19.6%)	53 (36.1%)	35 (46.1%)		
>4000	5 (5.4%)	16 (10.9%)	19 (25.0%)		
Medical payment method					
Urban and rural resident medical insurance	82 (89.1%)	81 (55.1%)	26 (34.2%)	55.060	<.001
Urban employee medical insurance	10 (10.9%)	66 (44.9%)	50 (65.8%)		
Disease duration					
<3 mo	24 (26.1%)	49 (33.3%)	53 (69.7%)	46.273	<.001
3 to 6 mo	31 (33.7%)	61 (41.5%)	18 (23.7%)		
>6 mo	37 (40.2%)	37 (25.2%)	5 (6.6%)		
Disease stage					
I	6 (6.5%)	29 (19.7%)	26 (34.2%)	60.044	<.001
II	33 (35.9%)	83 (56.5%)	44 (57.9%)		
III to IV	53 (57.6%)	35 (23.8%)	6 (7.9%)		
Course of chemotherapy					
1 to 2	28 (30.4%)	51 (34.7%)	34 (44.7%)	5.412	.248
3 to 5	42 (45.7%)	71 (48.3%)	28 (36.8%)		
6 to 8	22 (23.9%)	25 (17.0%)	14 (18.4%)		
Number of chemotherapy-related symptoms					
≤1	15 (16.3%)	54 (36.7%)	53 (69.7%)	66.380	<.001
2 to 3	41 (44.6%)	72 (49.0%)	19 (25.0%)		
≥4	36 (39.1%)	21 (14.3%)	4 (5.3%)		
Presence of chronic diseases					
Yes	21 (22.8%)	25 (17.0%)	13 (17.1%)	1.433	.489
No	71 (77.2%)	122 (83.0%)	63 (82.9%)		
Whether or not the patient had undergone surgery for breast cancer					
Yes	49 (53.3%)	105 (71.4%)	45 (59.2%)	8.705	.013
No	43 (46.7%)	42 (28.6%)	31 (40.8%)		

SRF = self-regulatory fatigue

### 3.4. Multivariate analysis of factors associated with SRF profiles

Multivariate logistic regression analyses were conducted using the 3 SRF profiles as dependent variables and age, monthly income (Chinese Yuan), medical payment method, disease duration, disease stage, number of chemotherapy-related symptoms, and whether or not breast cancer surgery was performed as independent variables. The results demonstrated that medical payment method, disease duration, disease stage, number of chemotherapy-related symptoms, and whether or not the patient had undergone surgery for breast cancer were factors potentially influencing SRF profiles (Table 5).

**Table 5:** Multivariate analysis of factors associated with SRF profiles.

Variables	*B*	SE	Wald	*P*	OR	95% CI
High SRF, cognitively weakened group VS Low SRF, behavioral stabilization group*						
Medical payment method						
Urban and rural resident medical insurance	1.920	0.498	14.840	<.001	6.823	(2.568–18.125)
Urban employee medical insurance†						
Disease duration						
<3 mo	−1.968	0.643	9.355	.002	0.140	(0.040–0.493)
3 to 6 mo	−1.368	0.655	4.365	.037	0.255	(0.071–0.919)
>6 mo†						
Disease stage						
I	−3.147	0.769	16.752	<.001	0.043	(0.010–0.194)
II	−2.024	0.598	11.465	.001	0.132	(0.041–0.426)
III to IV†						
Number of chemotherapy-related symptoms						
≤1	−2.149	0.710	9.168	.002	0.117	(0.029–0.469)
2 to 3	−0.576	0.684	0.709	.400	0.562	(0.147–2.150)
≥4†						
Moderate SRF, borderline group VS Low SRF, behavioral stabilization group*						
Disease duration						
<3 mo	−1.551	0.558	7.728	.005	0.212	(0.071–0.633)
3 to 6 mo	−0.695	0.588	1.399	.237	0.499	(0.158–1.579)
>6 mo†						
Disease stage						
I	−1.568	0.606	6.692	.010	0.208	(0.063–0.684)
II	−0.965	0.548	3.107	.078	0.381	(0.130–1.114)
III to IV†						
Whether or not the patient had undergone surgery for breast cancer						
Yes	0.770	0.370	4.322	0.038	2.159	(1.045–4.461)
No†						
High SRF, cognitively weakened group VS Moderate SRF, borderline group‡						
Medical payment method						
Urban and rural resident medical insurance	1.505	0.410	13.469	<.001	4.503	(2.016–10.059)
Urban employee medical insurance†						
Disease stage						
I	−1.578	0.571	7.626	.006	0.206	(0.067–0.633)
II	−1.059	0.354	8.950	.003	0.347	(0.173–0.694)
III to IV†						
Number of chemotherapy-related symptoms						
≤1	−1.170	0.471	6.175	.013	0.310	(0.123–0.781)
2 to 3	−0.636	0.384	2.739	.098	0.529	(0.249–1.124)
≥4†						

SRF = self-regulatory fatigue.

* Low SRF, behavioral stabilization group as a reference.

† Reference category.

‡ Reference category. Moderate SRF, borderline group as a reference.

### 3.5. Comparison of activation scores among patients with different SRF profiles

The mean (standard deviation) activation score of patients with breast cancer undergoing chemotherapy was 57.34 (8.82), which was at the third level overall, including 53 cases (16.8%) at the first level, 68 (21.6%) at the second level, 147 (46.7%) at the third level, and 47 (14.9%) at the fourth level. Comparisons of activation scores among patients with the 3 SRF profiles indicated that they differed significantly (*P < *.001) (Table 6).

**Table 6:** Comparison of activation scores among patients with different SRF profiles.

	High SRF, cognitively weakened group (n = 92)	Moderate SRF, borderline group (n = 147)	Low SRF, behavioral stabilization group (n = 76)	*F*	*P*	LSD
Total patient activation scores, M (SD)	50.59 (6.99)	57.66 (7.55)	64.88 (6.44)	83.707	<.001	Low SRF, behavioral stabilization group > Moderate SRF, borderline group*, Low SRF, behavioral stabilization group > High SRF, cognitively weakened group*, Moderate SRF, borderline group > High SRF, cognitively weakened group*

SRF = self-regulatory fatigue.

* *P* < .05.

## 4. Discussion

Three different potential categories of SRF were identified among patients with breast cancer undergoing chemotherapy. Medical payment method, disease duration, disease stage, number of chemotherapy-related symptoms, and whether or not the patient had undergone surgery for breast cancer were factors potentially influencing these SRF profiles. Further, comparison across SRF profiles indicated that they differed significantly according to patient activation score.

### 4.1. SRF heterogeneity among individuals with mammary carcinoma receiving chemotherapy

This study identified 3 SRF profiles in individuals with mammary carcinoma who received chemotherapy: a high SRF, cognitively weakened group; a moderate SRF, borderline group; and a low SRF, behavioral stabilization group. The high SRF, cognitively weakened group accounted for 29.2% of patients, who had higher scores on all dimensions of SRF-S than those with the other 2 profiles, with the highest scores on the cognitive dimensions, indicating that these patients had a heavier cognitive load. This may be due to the fact that illness and treatment not only make it difficult for patients to fulfill their family duties, but also increase financial pressure and caregiving needs, and patients are prone to guilt and self-blame, which leads to negative thoughts about their lives and interpersonal interactions.^[26,27]^ Our data suggest that patients in this group represent a key concern and that clinical teams can implement targeted interventions, starting with factors that can positively influence SRF. The moderate SRF, borderline group included 46.7% of patients, which was the largest proportion. This may be because most of the subjects included in this investigation were young and middle-aged patients, consistent with the trend toward breast cancer incidence at a younger age in China.^[28]^ This group of patients may have higher physical and mental energy reserves, greater recovery motivation, and better self-regulatory ability.^[29]^ However, due to the long duration of the disease and the fact that patients were at a critical stage of life, shouldering multiple responsibilities, such as family, work, etc., the majority of patients had moderate level SRF, suggesting that there remains room to improve SRF in this group of patients. Patients in the low SRF, behavioral stabilization group comprised 24.1% of total respondents. Our findings suggest that these patients had calmly accepted the reality of the disease and could effectively manage their behavior with less aggression. For this group, healthcare professionals should carefully assess their shortcomings and develop individualized strategies to help them maintain their low SRF levels.

### 4.2. Factors influencing SRF profiles

#### 4.2.1. Association of medical payment method with SRF

Our findings indicate that patients with urban and rural resident medical insurance were more prone to being a part of the high SRF, cognitively weakened group. This result is consistent with what Zhou reported.^[30]^ Breast cancer treatment is expensive. Individuals with urban and rural resident medical insurance are usually rural or urban residents with low incomes,^[31]^ and the reimbursement rate is relatively low, placing a greater financial burden on patients’ families. The economic basis motivates self-health management, suggesting that healthcare professionals should develop appropriate treatment plans based on the patient’s economic status to alleviate their financial stress as much as possible.

#### 4.2.2. Association of disease duration with SRF

Our data show that patients with shorter disease duration were more readily a part of the low SRF, behavioral stabilization group. Relative to those with a short disease duration, individuals with a longer disease duration experience more hospitalizations, longer periods of self-regulation, and use up more self-control resources as the disease progresses. Thus, these patients suffer from more severe SRF.^[32]^ It is recommended that healthcare professionals focus on patients with breast cancer undergoing chemotherapy who have longer disease duration, providing them with psychological therapy and assisting them in establishing a positive social support system to enhance their psychological resilience.^[33]^ Such approaches will enable patients to adopt effective coping strategies and decrease the depletion of their psychological resources.

#### 4.2.3. Association of disease stage with SRF

The results of the study showed that patients with later disease staging were more likely to be in the moderate SRF, borderline group, or high SRF, cognitively weakened group. A previous study confirmed that psychological distress in patients is associated with advanced disease stage.^[34]^ It may be because in the advanced stage of the tumor, the involvement of other parts of the body or vital organs, and the face of disease progression and uncertain prognosis, the patient’s physical and mental burden is heavier, and it is difficult for them to carry out self-regulation effectively.^[35]^ Therefore, caregivers should assess the SRF level in patients with advanced disease, effectively manage the symptoms of those with advanced tumors, provide health education for patients, and improve their poor psychological status.

#### 4.2.4. Association of the number of chemotherapy-related symptoms on SRF

Our data demonstrate that more patients with less than or equal to one of chemotherapy-related symptoms were included in the moderate SRF, borderline and low SRF, behavioral stabilization groups, consistent with previous studies.^[36]^ Patients with multiple chemotherapy-related symptoms have poorer physical function and decreased body regulation.^[37]^ They not only needed to face the challenges posed by the disease itself but also needed to manage the effects of their treatment-related symptoms, which increase the adverse emotional experiences of patients and aggravate SRF.^[38]^ These findings suggest that clinical staff should target nursing interventions according to the type and number of patient symptoms, as well as improve the ability of patients to manage chemotherapy-related symptoms.

#### 4.2.5. Association between whether or not the patient had undergone surgery for breast cancer and SRF

The results of this study indicate that patients who did not undergo breast cancer surgery were more likely to be categorized in the low SRF, behavioral stabilization group. After breast cancer surgery, the lack of secondary female characteristics makes patients dissatisfied with their image and prone to anxiety or depression.^[39]^ Surgical trauma leads to postoperative pain and other discomforts, affecting patients’ sleep quality and daily activities. Patients need regular functional exercises, and their SRF is heavy. Therefore, healthcare workers should assess patients’ physical and mental state who have undergone surgical treatment for breast cancer and intervene promptly. Healthcare professionals provide comprehensive treatment to patients with severe physical discomfort, explaining the importance of functional exercises and improving their confidence in recovery.

### 4.3. Variation in activation scores among patients with different SRF profiles

Breast cancer is a long-term health challenge that requires patients to actively cope with disease progression and participate in disease treatment and management to achieve improved health outcomes.^[40]^ Our results show that activation among patients with breast cancer undergoing chemotherapy was moderate, which aligns with the findings of Ma investigation^[41]^ and suggests that activation needs to be improved in such patients. In this study, we found that patients with varying levels of SRF had different patient activation scores, as follows scores were highest in the low SRF, behavioral stabilization group, followed by the moderate SRF, borderline group, and lowest in the high SRF, cognitively weakened group, implying that SRF may influence patient activation. The theory of ego depletion^[42]^ proposes that humans possess finite psychological energy and can only perform a limited number of self-control measures in the short term. In the disease management process, patients need to constantly self-regulate, for example, controlling emotions, adjusting lifestyles, and following medical advice. All of these activities consume patient psychological resources, leading to SRF, decreasing patient activation, and ultimately potentially affecting health outcomes. Healthcare professionals should prioritize patients with high levels of SRF, listen to their feelings, guide them to express their negative emotions in different ways, such as talking, writing, drawing, and work with patients to develop a specific and achievable health schedule, to enhance their self-management ability and confidence. For patients with less severe SRF, healthcare professionals should encourage them to maintain a good state of adaptation and further improve their confidence in actively coping with the disease. However, there have been few quantitative studies on the causal link between SRF and activation in individuals with breast cancer undergoing chemotherapy. More studies are needed to confirm this association in the future.

### 4.4. Limitations

This study investigated only 315 patients with breast cancer undergoing chemotherapy at 2 tertiary hospitals in Sichuan Province, China, which is a relatively small sample size. In the future, cross-regional and large-sample surveys can be conducted, to build on the results of this study. Second, this was a quantitative study, and qualitative or mixed studies could be conducted in the future, to facilitate deep analysis of patient SRF experiences and their influencing factors.

## 5. Conclusion

In this study, we used latent profile analysis to classify patients with breast cancer undergoing chemotherapy into 3 SRF profiles: high SRF, cognitively weakened; moderate SRF, borderline; and low SRF, behavioral stabilization groups. Medical payment method, disease duration, disease stage, number of chemotherapy-related symptoms, and whether or not the patient had undergone surgery for breast cancer were factors that potentially influenced SRF profile. Further, patient activation levels varied across SRF profiles. Our data suggest that medical personnel should target interventions to improve patient activation based on their different SRF profiles.

## Acknowledgments

The authors thank all the patients and staff who participated in this study.

## Author contributions

**Conceptualization:** Ying Yang, Li Li.

**Data curation:** Jing Zhao, Li Li.

**Formal analysis:** Jing Zhao, Yina Liu.

**Investigation:** Ying Yang.

**Supervision:** Li Li.

**Writing – original draft:** Ying Yang, Yina Liu.

**Writing – review & editing:** Ying Yang, Li Li.
